# Slug promoted vasculogenic mimicry in hepatocellular carcinoma

**DOI:** 10.1111/jcmm.12087

**Published:** 2013-07-01

**Authors:** Dan Sun, Baocun Sun, Tieju Liu, Xiulan Zhao, Na Che, Qiang Gu, Xueyi Dong, Zhi Yao, Rui Li, Jing Li, Jiadong Chi, Ran Sun

**Affiliations:** a Department of Pathology, Tianjin Medical UniversityTianjin, China; b Department of Pathology Tianjin Cancer Hospital, Tianjin Medical UniversityTianjin, China; c Department of Pathology Tianjin General Hospital, Tianjin Medical UniversityTianjin, China

**Keywords:** Slug, cancer stem cells, vasculogenic mimicry, hepatocellular carcinoma

## Abstract

Vasculogenic mimicry (VM) refers to the unique capability of aggressive tumour cells to mimic the pattern of embryonic vasculogenic networks. Epithelial–mesenchymal transition (EMT) regulator slug have been implicated in the tumour invasion and metastasis of human hepatocellular carcinoma (HCC). However, the relationship between slug and VM formation is not clear. In the study, we demonstrated that slug expression was associated with EMT and cancer stem cell (CSCs) phenotype in HCC patients. Importantly, slug showed statistically correlation with VM formation. We consistently demonstrated that an overexpression of slug in HCC cells significantly increased CSCs subpopulation that was obvious by the increased clone forming efficiency in soft agar and by flowcytometry analysis. Meantime, the VM formation and VM mediator overexpression were also induced by slug induction. Finally, slug overexpression lead to the maintenance of CSCs phenotype and VM formation was demonstrated *in vivo*. Therefore, the results of this study indicate that slug induced the increase and maintenance of CSCs subpopulation and contributed to VM formation eventually. The related molecular pathways may be used as novel therapeutic targets for the inhibition of HCC angiogenesis and metastasis.

## Introduction

A non-angiogenesis-dependent pathway, in which tumours can feed themselves, has been reported [1]. The process by which a vessel is formed from tumour cells is called vasculogenic mimicry (VM). The presence of VM was associated with a high tumour grade, invasion and metastasis, and short survival [2–5].

Our previous studies have implicated transcriptional factors Twist1 and epithelial–mesenchymal transition (EMT)in the formation of VM by human hepatocellular carcinoma (HCC) cells *in vivo* and *in vitro* [6]. It has been suggested that there is a direct link between the EMT and the gain of epithelial stem cell properties. The recent study found that EMT could promote the property of stemness in normal cells as well as cancer cells [7–9].

Slug (SNAI2), belonging to zinc-finger transcription factors, was reported to be an essential mediator of Twist1-induced EMT and metastasis [10]. CSCs have been shown to not only promote tumour angiogenesis [11] but also have the ability of transdifferentiation into endothelial cells. In recent research, slug overexpression was associated with CSC ‘stemness’ behaviour [12, 13]. Slug not only can regulate the cancer stem cell immunophenotype but also can mediate radioresistance and chemoresistance by inducing cancer stem-like properties [14].

However, the relationship of slug, CSCs phenotype and VM in HCC is currently unknown. In this study, we try to identify the potential contribution of slug to tumour VM formation and thus provide novel therapeutic strategies for HCC.

## Materials and methods

### Patient samples

Through the Tumor Tissue Bank of Tianjin Cancer Hospital, tissue specimens were obtained from 113 patients who underwent hepatectomy for HCC between 2001 and 2010. The diagnoses of these HCC samples were verified by pathologists. Detailed pathological and clinical data were collected for all samples including Edmondson tumour grade, metastasis and survival duration. Tissue collection and analysis in this study were approved by the Ethical Committee of Tianjin Medical University, China.

### Immunohistochemical and histochemical double-staining methods

The assay was performed as previously described [5, 6].

### Quantitation of slug, CD90, E-cadherin, vimentin, VEGF and VE-cadherin staining

At least 10 power fields were chosen per case and >500 cells were counted for each power field. Scoring system was modified and used according to evaluation standard [15]. The percentage of the staining cells (P) was scored as follows: 0 (negative staining), 1 (≤10% of cells), 2 (10–50%) and 3 (≥50%) for slug quantitation. 0 (negative staining), 1 (≤25% of cells), 2 (≤50%) and 3 (>50%) for CD90, E-cadherin, vimentin, VEGF and vascular endothelial (VE)-cadherin quantitation respectively. Staining intensity (I) was graded as follows: 0 (no staining), 1 (weak staining), 2 (moderate staining), 3 (intense staining). Samples in each power field were evaluated for both factors, *i.e*. P plus I. The scoring of each case was a mean value of chosen power fields. The cases with scoring ≥3 were identified as positive expression.

### Cell culture and stable cell lines

Human liver cancer cell lines (HepG2, SMMC7221, Bel7402, Huh-7) were obtained from American Type Culture Collection (ATCC, USA), and the Cell Bank of the Chinese Academy of Medical Sciences (Beijing, China). Transfection in HepG2 and Huh-7 cells was performed with lipofectamine 2000 reagent, and clones selected by G418.

### Expression plasimids

Full-length Slug complementary DNA (cDNA) was generated by normal human embryo total cDNA, and digested with XhoI/EcoRI and subcloned into pcDNA3.1 vectors. The resulting constructs were confirmed by DNA sequencing.

### Western blot analysis and immunofluorescence staining

See supplementary data.

### Flow cytometry

Indirect labelling method was performed. After trypsinized, cells were fixed with 75% ethanol, incubated with primary antibodies CD133 (Santa Cruz, Dallas, TX, USA), CD90 (Gene Tex, Irvine, CA, USA) at 37°C for 1 hr. Subsequently, the cells were stained with fluorescein isothiocyanate (FITC) or tetramethyl rhodamine isothiocyanate–conjugated mouse and rabbit immunoglobulin G antibody. All flow cytometric data were analysed with CFlow software.

### Invasion

Cell migration assay was performed with Transwell cell culture inserts (Invitrogen, Carlsbad, CA, USA) according to the manufactory's instruction.

### Soft agar colony formation assay

The assay was performed as previously described [16].

### 3D cultures

The assays were performed as previously described [5, 6].

### Xenograft

All animal work had been conducted according to the guidelines of Tianjin Medical University, China. Male BALB/c nude mice, 5 weeks of age, were purchased from Beijing, China. 5 × 10^6^ viable cells/0.1 ml of PBS were injected into the armpit of 20 mice with a 26-gauge needle. For 30 days, the mice were monitored and tumour sizes were measured weekly using a calliper. The tumour volume (TV) was calculated by the following formula: TV = 1/2 × *a* × *b*^2^ (in which *a* is the length and *b* is the width of tumour).

### Statistical analysis

The data analysis was performed with the SPSS16.0 (SPSS, Chicago, IL, USA) software package. All P values were two-sided, and statistical significance was set at *P* = 0.05.

## Results

### Expression of slug in correlation with cancer stem cell phenotype in human HCC tissue

**Fig. 1:**
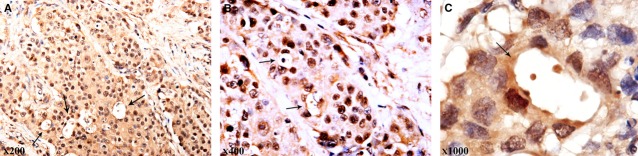
Expression of slug in human hepatocellular carcinoma (HCC) tissue. (**A**–**C**) Slug-positive expression in primary HCC tissue was identified in the cytoplasm as well as in the nucleus of cancer cells. Black arrows showed slug-positive tumour cells either could form vascular vessels or involved in mosaic vessels with endothelial cells including red blood cells.

Based on the criteria Hotz *et al*. [17] established with minor modification, slug in primary HCC tissue was identified in the cytoplasm as well as in the nucleus of cancer cells (Fig. 1A–C). The percentage of the positive cells ≥10% was considered as slug-positive case. Thirty nine (35.5%) of 113 cases displayed slug overexpression. By quantitation of slug expression, the scoring of slug staining was 3.35 ± 0.09 and 1.43 ± 0.07 in slug-positive group and -negative group respectively (*P* = 0.000). The scoring <3 for slug expression in HCC tissue was considered as endogenous slug level in aggressive HCC cells. Interestingly, we observed that slug-positive tumour cells had close relationship with vascular vessel formation. Slug-positive tumour cells either could form vascular vessels or involved in mosaic vessels with endothelial cells (Fig. 1A–C arrow), suggesting that slug played an important role in tumour vasculature. Slug had been shown to induce EMT, a fundamental mechanism of embryogenesis and progressive disease. Then, we next examined EMT makers E-cadherin and vimentin expression (Figure S1A–D). 74.4% (29/39) cases of slug overexpression showed a reduced E-cadherin expression pattern (Figure S1B), whereas 41.9% (31/74) cases of low slug expression had a reduced pattern, with a statistically significant difference (χ^2^ = 10.810, *P* = 0.001). The scoring of E-cadherin was 2.28 ± 0.25 in slug-positive group and 3.19 ± 0.24 in slug-negative group (*P* = 0.019). Similarly, more patients with slug overexpression displayed vimentin expression (28.2%, 11/29; Figure S1D), whereas low slug expression show vimentin expression in only 12.2% (9/74) cases (χ^2^ = 4.513, *P* = 0.034). The scoring of vimentin was 2.77 ± 0.19 in slug-positive group and 2.11 ± 0.14 in slug-negative group (*P* = 0.006). Statistically significant correlations were found among E-cadherin, vimentin and slug expression (*r* = 0.309, *P* = 0.001 for slug and E-cadhern; *r* = 0.200, *P* = 0.034 for slug and vimentin). Slug overexpression significantly correlated with reduced E-cadherin expression and increased vimentin expression. Similar results were obtained when slug, E-cadherin and vimentin expression was quantified. Slug expression showed a lower scoring in E-cadherin-positive group (1.81 ± 0.14) than -negative group (2.35 ± 0.14) (*P* = 0.009), and a higher scoring in vimentin-positive group (2.30 ± 0.29) than -negative group (2.05 ± 0.11), although no significance was found (*P* = 0.367).

**Fig. 2:**
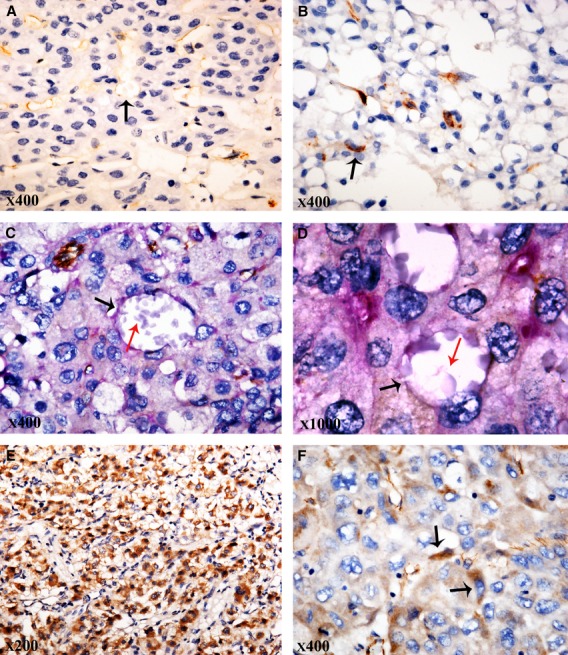
Expression of slug in correlation with cancer stem cell phenotype and vasculogenic mimicry (VM) in human hepatocellular carcinoma (HCC) tissue. (**A**) Hepatocellular cancer cells arranged in tubular structure showed apical/endoluminal cell surface CD90 staining. Black arrow showed that CD90^+^ tumour cells were involved in blood vessel formation. (**B**) CD90 positivity was observed as abundant cytoplasmic and membrane staining in HCC specimen. (**C**–**D**) Vasculogenic mimicry present in HCC tissue by CD31/PAS double staining. (**E**) VEGF positivity present in the cytoplasm in HCC tissue with slug-positive expression. (**F**) Vascular endothelial (VE)-cadherin positivity present in the cytoplasm in HCC tissue and tumour cells with spindle morphology displayed stronger VE-cadherin expression.

Recent reports [18–20] indicate that the emergence of CSCs occurs, in part, as a result of EMT. There is a direct link between the EMT program and the gain of epithelial stem cell properties. We next examined the existence of CSCs in HCC tissues. CD90 is identified as specific antigenic markers for HCC stem cells. Two distinct CD90 staining patterns were observed. The first pattern was observed in the cancer cells that formed tubular structures. Hepatocellular cancer cells arranged in tubular structure showed apical/endoluminal cell surface CD90 staining (Fig. 2A). We observed that CD90^+^ tumour cells of the pattern were involved in vessels formation (Fig. 2A arrow). The second pattern was the abundant cytoplasmic and membrane positivity of CD90 staining (Fig. 2B). The percentage of CD90^+^ cells in the total tumour cells ranged from 5% to 30% in the positive case. Immunohistochemistry of 113 HCC tissues showed a moderate-to-strong CD90 expression in 22 (19.5%) cases. More patients with high slug expression displayed high CD90 expression (30.8%, 12/39), whereas low slug expression showed 1.4% (10/74) cases with CD90-positive expression. By the quantitation of slug and CD90 expression, slug staining showed a higher scoring in CD90-positive group (2.59 ± 0.23) than -negative group (1.98 ± 0.11; *P* = 0.019), and the scoring of CD90 was 3.15 ± 0.16 in slug-positive group and 2.26 ± 0.11 in slug-negative group (*P* = 0.000). By statistical analysis, there is correlation between slug and CD90 expression (*r* = 0.207, *P* = 0.028).

### Expression of slug in correlation with VM in human HCC tissue

By CD31 and periodic acid-Schiff (PAS) histochemical and immunohistochemical double staining, typical microvessels showed positive reaction for CD31 on their luminal surface and PAS-positive reaction in their wall. Based on our previous studies [5, 6], CD31/PAS double staining was used to identify VM in HCC tissue. CD31-negative, PAS-positive vascular-like patterns containing red blood cells, which formed by HCC cells, were deemed VM. HCC cells formed extracellular matrix–rich channels (PAS-positive), with the absence of necrosis and inflammatory cells infiltrating around the channels. VM channels were not lined by endothelial cells as demonstrated by the lack of CD31 (brown) staining (Fig. 2C–D). The highest VM area was identified and individual VM channel counts were made on a 200× field. At least 40 power fields were chosen per case. By VM channel counting, the median value showed 4.20 ± 0.80 in slug-positive expression and 0.18 ± 0.12 in slug-negative expression. There were significant differences between the two groups (*t* = 6.662, *P* = 0.000). Therefore, the presence of VM was closely associated with slug-positive expression.

VEGF is one of the most potent and specific angiogenic factors of tumour-induced angiogenesis. Cancer stem cell-like cells expressed much higher levels of VEGF and formed more tumours with more blood vessels than cancer cells that did not have stem cell characteristics. Accordingly, VEGF creates a perivascular niche for CSCs and stimulates cancer stemness and renewal [21]. By IHC, we found that VEGF expression was present in 61.1% (69/113) HCC samples (Fig. 2E). VEGF expression displayed a close relationship with CSCs phenotype (CD90-positive expression) by statistical analysis (*r* = 0.255, *P* = 0.006). Meantime, VEGF showed a higher expression in slug positive than in slug negative (χ^2^ = 4.429, *P* = 0.035) and displayed significant correlation with slug overexpression by statistical analysis (*r* = 0.198, *P* = 0.036). In addition, by the quantitation of slug and VEGF expression, the scoring of VEGF was 4.26 ± 0.18 in slug-positive group and 3.55 ± 0.16 in slug-negative group (*P* = 0.008), and slug staining showed a higher scoring in VEGF-positive group (2.26 ± 0.13) than -negative group (1.84 ± 0.17; *P* = 0.048).

Vascular endothelial-cadherin was transmembrane glycoprotein that was expressed in the adherens junctions between vascular endothelial cells (EC). Vascular endothelial-cadherin was essential during embryonic angiogenesis. Vascular endothelial-cadherin expression by EC played an important role in regulating vascular morphology and stability. In addition, VE-cadherin exclusively expressed by highly aggressive melanoma cells was critical in melanoma VM. In our study, 48 (42.5%) of 113 cases showed VE-cadherin expression in HCC specimen. By statistical analysis, VE-cadherin was associated with VM formation (*r* = 0.399, *P* = 0.000), suggesting that it could be a VM mediator in HCC. We observed that tumour cells with spindle morphology displayed stronger VE-cadherin expression (Fig. 2F), suggesting that epithelial–endothelial transition (EET) existed in HCC. Importantly, VE-cadherin expression was positively related with slug expression (*r* = 0.205, *P* = 0.030). By the quantitation of slug and VE-cadherin expression, the scoring of VE-cadherin was 3.28 ± 0.26 in slug-positive group and 2.12 ± 0.22 in slug-negative group (*P* = 0.002), and slug staining showed a higher scoring in VE-cadherin-positive group (2.35 ± 0.16) than -negative group (1.91 ± 0.13; *P* = 0.033).

### Constitutive activation of slug-induced EMT, developed CSCs phenotype and VM in HCC cells *in vitro*

**Fig. 3:**
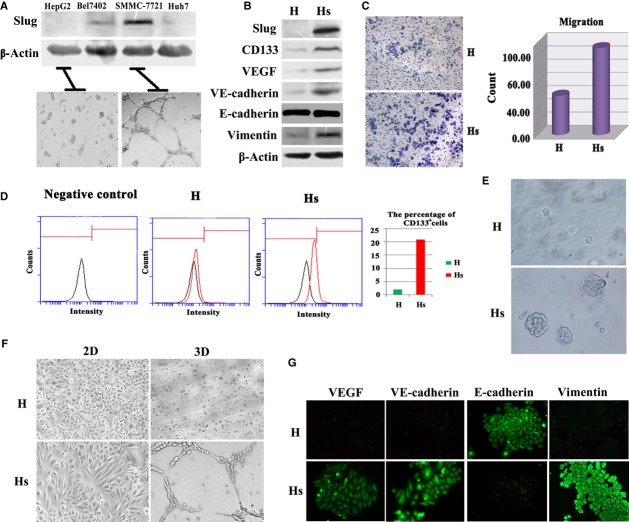
Constitutive activation of slug-induced epithelial–mesenchymal transition, developed CSCs phenotype and vasculogenic mimicry (VM) in human hepatocellular carcinoma (HCC) cells *in vitro*. (**A**) HepG2 and huh7 had a low-level slug expression in contrast with the SMMC-7721, which presented a high level. HepG2 and huh7 cells with slug low expression could not form typical pipe-like structures; in contrast, SMMC-7721 with high slug expression could form VM. (**B**) HepG2 cells transfected with slug cDNA expressed higher levels of slug protein, CD133+ expression, VEGF, vascular endothelial (VE)-cadherin and vimentin expression. (**C**) An increase in cell invasion was observed in the HepG2-slug cells (HS) when compared with HepG2-control (H) cells. (**D**) Flowcytometry analysis showed that HepG2-slug developed a subpopulation (∼21.4%) of CD133+ or CD90+ CSCs phenotype, whereas parental HepG2 cells displayed a CD133− or CD90− phenotype. (**E**) HepG2-slug exhibited higher colony-forming efficiency and formed more colonies than HepG2-control cells. (**F**) HepG2-slug formed typical pipe-like structures within the 3D Matrigel medium with exogenous slug expression. (**G**) By immunofluorescence, VEGF and VE-cadherin expression was detected in the cytoplasm of HepG2-slug (Hs). E-cadherin expression was identified in the cell membrane and less intensive in the cytoplasm in Hs. Vimentin expression was displayed in the cytoplasm in Hs.

We compared the level of protein of slug expression and found that slug was differentially expressed in various HCC cell lines by western blotting. We found that HepG2 and huh7 had a low-level slug expression in contrast with the SMMC-7721, which presented a high level (Fig. 3A). We then used a well-established Matrigel culture for investigating VM formation. Interestingly, HepG2 and huh7 cells with low slug expression could not form typical pipe-like structures; in contrast, SMMC-7721 with high slug expression could form VM (Fig. 3A).

In this study, HepG2 and huh7 cells transfected with slug cDNA expressed higher levels of slug protein than the untransfected cells as analysed by western blot (Fig. 3B). To determine the endogenous slug expression in HepG2 cells, the band intensities of slug or β-actin was quantified by Image J software. The protein ratios were calculated by dividing the intensity of slug band by the intensity of β-actin band. The slug/β-actin ratio was 0.49 in HepG2 cells, which suggested that there was endogenous slug expression in HepG2 cells. In contrast, the slug/β-actin ratio was 1.14 in HepG2 cells with slug transfectant, suggesting that the exogenous slug expression was much higher than the endogenous slug expression. In the invasion assay presented in Figure 3C, an increase in cell invasion was observed in the HepG2-slug cells (HS) when compared with HepG2-control cells (H). HepG2-slugs showed spindle morphology in 2D culture (Fig. 3F) compared with control cells, suggesting that an EMT phenotype might be induced by slug introduction. We then analysed the effect of slug overexpression on EMT phenotype in HepG2 and huh7 cells. Western blot analysis and immunofluorescence showed that the expression of epithelial marker E-cadherin was downregulated and mesenchymal marker vimentin was upregulated when compared with the negative vector controls (Fig. 3B, G and Figure S2A).

Meantime, Flowcytometry analysis showed that HepG2 and huh7 cells with slug overexpression developed a subpopulation (∼21.4%) of CD133^+^ or CD90^+^ CSCs phenotype, while parental HepG2 and huh7 cells displayed a CD133^−^ or CD90^−^ phenotype (Fig. 3D and Figure S2B). To further demonstrate HepG2-slug developed the CSCs-like subpopulation that was not seen in the cultured HepG2 cells, soft agar colony formation assays were performed. The colonies were scored to determine the colony-forming efficiency (CFE) after culture. Our studies showed that HepG2-slug exhibited higher CFE and formed more colonies than HepG2-control cells (Fig. 3E). HepG2-slug cells had a CFE of 12.8 ± 1.46%, whereas control HepG2 cells had a lower CFE of 3.00 ± 1.71% (*t* = 6.031, *P* = 0.000). The HepG2-slug cells showed ∼fourfold increase in CFE when compared with that of HepG2-control cells, suggesting that HepG2-slug cells have a higher proliferative potential and might developed more CSCs subpopulation.

Remarkably, HepG2-slug and huh7-slug cells displayed a higher vasculogenic capacity than control cells. HepG2 and Huh7 cells without VM formation ability formed typical pipe-like structures within the 3D Matrigel medium with exogenous slug expression (Fig. 3F and Figure S2C). Our results provided further support for the possible role of slug in promoting VM formation. In addition, HepG2-slug and huh7-slug acquired higher endothelial cell marker VE-cadherin and VEGF expression than HepG2-control cells (Fig. 3B, G and Figure S2A). The expression of VE-cadherin and VEGF in HepG2-slug and huh7-slug cells suggested that HepG2-slug and huh7-slug cells with more CSCs subpopulation might have the capacity of transdifferentiation to differentiate into endothelial cells and acquire endothelial cell phenotype. Therefore, HepG2-slug and huh7-slug cells were more potent in vascular channel formation.

### HepG2-slug xenografts maintained CSCs and EMT phenotype, augmented vasculogenic mimicry

**Fig. 4:**
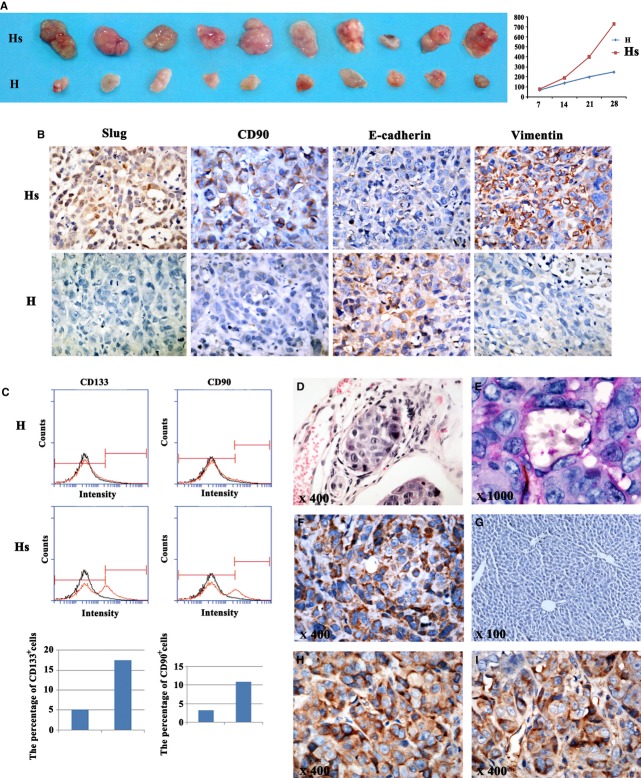
HepG2-slug (Hs) xenografts maintained CSCs and epithelial–mesenchymal transition phenotype, augmented vasculogenic mimicry. (**A**) Xenografts showed a higher rate of tumour growth in Hs as compared with the parental HepG2 (H) cells. (**B**) Positive slug and CD90 expression, the downregulated E-cadherin expression and upregulated vimentin expression present in Hs xengraft. (**C**) Flowcytometry analysis showed that CD133+ and CD90+ phenotype were present in Hs xenograft. (**D**) There was cancer embolus present in blood vessels in Hs xenograft. (**E**) Vasculogenic mimicry (VM) was present in Hs xenograft. (**F**) Monoclonal Rabbit anti-Human HLA-DR antibody was used to identify human cell and positive staining was in the cytoplasm. Black arrow showed that HLA-DR-positive cells had been incorporated into VM channels. (**G**) The mouse liver tissue showed HLA-DR negative. (**H** and **I**) VEGF and VE-cadherin showed higher expression in Hs xenograft.

To characterize the molecular mechanisms linking slug activation with tumour progression *in vivo*, we developed a xenograft model of human HCC progression employing the HepG2-slug cells and parental HepG2 cells as control. Following subcutaneously transplant of HepG2-slug cells into nude mice, xenografts showed a higher rate of tumour growth as compared with the parental HepG2 cells (Fig. 4A). Although the HepG2-slug xenografts did not give rise to spontaneous distant organ metastases by 28 days of tumour growth, there was cancer embolus present in blood vessels (Fig. 4D). In contrast, HepG2 cancer xenografts that typically fail to grow vigorously in nude mice displayed low propensity to invasive to vessels. Remarkably, after *in vivo* growth, HepG2-slug xenografts maintained CSCs phenotype with the presence of subpopulations of cancer cells harbouring a CD133+ or CD90+ phenotype (Fig. 4B and C) that was not seen in the HepG2 xenografts where the predominant phenotype was CD133− or CD90−. Meantime, EMT phenotype was also persisted in HepG2-slug xenograft (Fig. 4B).

Vasculogenic mimicry was identified by endomucin/PAS double staining (Fig. 4E). Monoclonal Rabbit anti-Human HLA-DR antibody was used to identify human cell and we found that HLA-DR-positive cells had been incorporated into VM channels (Fig. 4F black arrow). In contrast, the mouse liver tissue showed HLA-DR negative (Fig. 4G).

By VM channel counting, the median value showed 7.00 ± 0.37 in HepG2-slug xenograft and 1.10 ± 0.35 in HepG2 xenograft. There were significant differences between the two groups (*t* = 11.696, *P* = 0.000). Therefore, the results demonstrated that slug overexpression contributed to VM formation *in vivo*.

Likewise, VEGF and VE-cadherin showed higher expression (Fig. 4H and I) in HepG2-slug compared with HepG2 xenograft, suggesting that the increased CSCs subpopulation induced by slug overexpression constitutively expressed VEGF and VE-cadherin, played an important role in contributing VM formation *in vivo*.

To evaluate whether endogenous slug plays any role in HCC cells with high slug expression, we knocked down slug expression in the SMMC-7721 cells with VM formation ability using slug siRNA. The concomitant decrease in the slug protein level in the slug siRNA-treated cells was evident from the Western blot data. Remarkably, with slug knock down, SMMC-7721 cells could not form pipe-like structure on Matrigel (VM, Figure S3), suggesting that slug played an important role in VM formation. Then, SMMC-7221 and SMMC-7721 cells with slug silencing were injected into the armpit of nude mice. SMMC-7721 cells with slug silencing showed the reduced VE-cadherin, VEGF and mesenchymal marker vimentin expression and the increased E-cadherin expression, the restored CD90^-^ non-CSCs phenotype and the inhibition of VM formation (Figure S3).

## Discussion

Tumour growth and invasion are dependent on a persistent blood supply; therefore, the capability of generating neovessels through diverse mechanisms is associated with its malignant potential in tumour [22–27]. Vasculogenic mimicry means that tumour cells have a larger plasticity and can make space for blood inflow when the more aggressive tumour need more blood during the process of tumour growth and invasion. Vasculogenic mimicry is easy to be found in the more aggressive tumour. After the blood supply for tumour growth and invasion had been satisfied by VM, the endothelial cells can grow into the space made by tumour cells and then angiogenesis and vasculogenesis are induced consequently. This study demonstrates a novel role of transcriptional repressor slug in the development of VM in HCC. The formation of fluid-conducting networks by non-endothelial cells has been described for melanomas, hepatocellular, breast, colon and prostate carcinomas as a result of VM, which is a feature associated with a pluripotent gene expression pattern in aggressive tumour cells. In our study, the vigorous VM was present in slug overexpression patients. Slug overexpression could promote VM, suggesting that HCC cells with slug overexpression have a more aggressive phenotype and a bigger capability of growth and invasion.

Our data showed that a correlation between expression levels of slug and decreased E-cadherin expression and increased vimentin expression was obvious in these human specimens, thus indicating that slug is sufficient to induce EMT. The induction of EMT can generate a population with stem cell characteristics from well-differentiated epithelial cells and cancer cells [28–30]. In our study, we found that slug overexpression not only related to EMT but also related to CSCs phenotype. Our study suggested that slug overexpression might lead to poor prognosis through promoting VM that could be induced by an EMT-like conversion and an increased population of cells displaying CSC markers demonstrating the plasticity of epithelial cells.

Ectopic slug overexpression *in vitro* also showed that an EMT phenotype was induced. Meantime, our data showed that EMT and CSCs phenotype induced by slug overexpression could be linked to each other. By overexpressing the major EMT regulator slug in HepG2 cells, we induced an EMT-like state (decreased E-cadherin and increased expression of mesenchymal markers) and showed that these cells had an increased ability to self-renew and a higher CFE, a property normally associated with epithelial cancer stem cells. Remarkably, there was significant difference for the formation of vascular network on Matrigel between HepG2-slug and parental HepG2. With the ectopic introduction of slug with up-regulation in HepG2 dells, the cells with slug transfectant formed typical pipe-like structures, although there was a lack of tube formation in the parental cells. Our study provided further support for the role of slug in promoting vascular channels formation.

Recent studies have suggested that tumour cells might be the progenitor for tumour vasculature [31, 32]. Most likely, tumour cells that display stem cell–like characteristics can undergo asymmetric cell division giving rise to tumour cells that trigger angiogenic programmes [33]. CSCs might have the unique ability to express an endothelial phenotype and to form vessel-like networks, ‘mimicking’ the pattern of embryonic vascular networks [34, 35]. Our results indicated that the mechanism of VM formation induced by slug overexpression was closely related to an increase in CSCs subpopulation generated from EMT. Then, the increased CSCs can transdifferentiate into different phenotype, they express angiogenic and vasculogenic markers such as VEGF and VE-cadherin and they are able to organize pseudovascular network.

The development of EMT, stemness, a CSCs phenotype and VM formation *in vivo* was also associated with an increase in slug expression indicating that slug were responsible for the maintaining of CSCs phenotype during *in vivo* growth. Meantime, VE-cadherin and VEGF expression was also increased in HepG2-slug xenograft and it suggested that the process of EET we named before occurred with the non-CSCs/CSCs switch *in vivo*. The EET promoted by slug overexpression can be used for vascular structure. CSCs in HepG2-slug *in vivo* can further differentiate into endothelial cell-like tumour cells to participate in the construction of tumour microcirculation. In addition, cancer stem–like cells might also directly contribute to the tumour angiogenesis by converting to endothelial cell [36, 37]. Our study showed that the tumours in HepG2-slug xenograft presented more vascular vessels of human tumour cell origin than HepG2 xenograft. It demonstrated that slug overexpression could contribute to tumour angiogenesis *in vivo*, especially contribute to vascular vessels formation of tumour cell origin.

Therefore, in a word, this study demonstrated that slug promote VM in HCC by the induction of EMT, pluripotency and CSCs-like phenotype *in vitro*, *in vivo* and in HCC patients. Vasculogenic mimicry represents an important survival mechanism contributing to the failure of currently available angiogenesis inhibitors to fully effect tumour eradication. Thus, our study suggested that the molecular target of slug might act on specific CSCs subpopulation and opened of course interesting new therapeutic perspectives for the treatment of HCC.

## Supporting information

**Data S1** Supplementary data.

**Figure S1** Cases of slug overexpression showed a reduced E-cadherin expression pattern and an increased vimentin expression pattern in HCC patients.

**Figure S2** Huh7 cells transfected with slug showed CD133^+^ and CD90^+^ phenotype, EMT phenotype, VEGF and VE-cadherin expression and VM formation.

**Figure S3** (A) The decreased expression of slug protein in the slug siRNA-treated SMMC-7721 cells (S: SMMC-7721; si S: SMMC-7721 with slug silencing) was evident from the Western blot data. Slug silencing inhibited tube formation (VM) in SMMC-7721 cells *in vitro*. (B-Q) SMMC-7721 with slug silencing xenografts reversed CSCs and EMT phenotype, inhibited vasculogenic mimicry. Positive slug (B), CD90 expression (D), the loss of E-cadherin expression (F), positive vimentin expression (H), higher VE-cadherin (L) and VEGF (N) expression present in S (SMMC-7721) xenograft. In contrast, the decreased slug (C) and CD90 expression (E), the increased E-cadherin expression (G), and the decreased vimentin (I), VE-cadherin (M) and VEGF (O) present in si S (SMMC-7721 with slug silencing) xenograft. There was VM (black arrow indicated VM channel; green arrow indicated red blood cell; red arrow indicated endomucin-positive blood vessel) present in SMMC-7221 xenograft (J). However, in si S (SMMC-7721 with slug silencing) xenograft, no VM channels were found (K). HLA-DR-positive tumour cells formed VM channel (black arrow indicated VM; red arrow indicated red blood cell) in SMMC-7221 xenograft (P). However, in si S (SMMC-7721 with slug silencing) xenograft, no VM channels were found (Q).

## References

[1] Dome B, Hendrix MJ, Paku S (2007). Alternative vascularization mechanisms in cancer: pathology and therapeutic implications. Am J Pathol.

[2] Sun B, Zhang S, Zhang D (2006). Vasculogenic mimicry is associated with high tumor grade, invasion and metastasis, and short survival in patients with hepatocellular carcinoma. Oncol Rep.

[3] Kirschmann DA, Seftor EA, Hardy KM (2012). Molecular pathways: vasculogenic mimicry in tumor cells: diagnostic and therapeutic implications. Clin Cancer Res.

[4] Wang SY, Yu L, Ling GQ (2012). Vasculogenic mimicry and its clinical significance in medulloblastoma. Cancer Biol Ther.

[5] Sun T, Sun BC, Zhao XL (2011). Promotion of tumor cell metastasis and vasculogenic mimicry by way of transcription coactivation by Bcl-2 and Twist1: a study of hepatocellular carcinoma. Hepatology.

[6] Sun T, Zhao N, Zhao XL (2010). Expression and functional significance of Twist1 in hepatocellular carcinoma: its role in vasculogenic mimicry. Hepatology.

[7] Mani SA, Guo W, Liao MJ (2008). The epithelial-mesenchymal transition generates cells with properties of stem cells. Cell.

[8] Kasimir-Bauer S, Hoffmann O, Wallwiener D (2012). Expression of stem cell and epithelial-mesenchymal transition markers in primary breast cancer patients with circulating tumor cells. Breast Cancer Res.

[9] Sarrio D, Franklin CK, Mackay A (2012). Epithelial and mesenchymal subpopulations within normal basal breast cell lines exhibit distinct stem cell/progenitor properties. Stem Cells.

[10] Casas E, Kim J, Bendesky A (2011). Snail2 is an essential mediator of Twist1-induced epithelial mesenchymal transition and metastasis. Cancer Res.

[11] Bao S, Wu Q, Sathornsumetee S (2006). Stem cell-like glioma cells promote tumor angiogenesis through vascular endothelial growth factor. Cancer Res.

[12] Bhat-Nakshatri P, Appaiah H, Ballas C (2010). SLUG/SNAI2 and tumor necrosis factor generate breast cells with CD44^+^/CD24^−^ phenotype. BMC Cancer.

[13] Zhu LF, Hu Y, Yang CC (2012). Snail overexpression induces an epithelial to mesenchymal transition and cancer stem cell-like properties in SCC9 cells. Lab Invest.

[14] Kurrey NK, Jalgaonkar SP, Joglekar AV (2009). Snail and slug mediate radioresistance and chemoresistance by antagonizing p53-mediated apoptosis and acquiring a stem-like phenotype in ovarian cancer cells. Stem Cells.

[15] Bittner M, Meltzer P, Chen Y (2000). Molecular classification of cutaneous malignant melanoma by gene expression profiling. Nature.

[16] Liu TJ, Sun BC, Zhao XL (2013). CD133 + cells with cancer stem cell characteristics associates with vasculogenic mimicry in triple-negative breast cancer. Oncogene.

[17] Hotz B, Arndt M, Dullat S (2007). Epithelial to mesenchymal transition: expression of the regulators snail, slug, and twist in pancreatic cancer. Clin Cancer Res.

[18] Raimondi C, Gianni W, Cortesi E (2010). Cancer stem cells and epithelial-mesenchymal transition: revisiting minimal residual disease. Curr Cancer Drug Targets.

[19] Battula VL, Evans KW, Hollier BG (2010). Epithelial-mesenchymal transition-derived cells exhibit multilineage differentiation potential similar to mesenchymal stem cells. Stem Cells.

[20] Singh A, Settleman J (2010). EMT, cancer stem cells and drug resistance: an emerging axis of evil in the war on cancer. Oncogene.

[21] Beck B, Driessens G, Goossens S (2011). A vascular niche and a VEGF-Nrp1 loop regulate the initiation and stemness of skin tumours. Nature.

[22] Cao Y (2010). Off-tumor target–beneficial site for antiangiogenic cancer therapy?. Nat Rev Clin Oncol.

[23] Xue Y, Lim S, Yang Y (2012). PDGF-BB modulates hematopoiesis and tumor angiogenesis by inducing erythropoietin production in stromal cells. Nat Med.

[24] Cao Y, Arbiser J, D'Amato RJ (2011). Forty-year journey of angiogenesis translational research. Sci Transl Med.

[25] Cao Y, Langer R (2010). Optimizing the delivery of cancer drugs that block angiogenesis. Sci Transl Med.

[26] Cao R, Xue Y, Hedlund EM (2010). VEGFR1-mediated pericyte ablation links VEGF and PlGF to cancer-associated retinopathy. Proc Natl Acad Sci USA.

[27] Cao R, Ji H, Feng N (2012). Collaborative interplay between FGF-2 and VEGF-C promotes lymphangiogenesis and metastasis. Proc Natl Acad Sci USA.

[28] Pirozzi G, Tirino V, Camerlingo R (2011). Epithelial to mesenchymal transition by TGFbeta-1 induction increases stemness characteristics in primary non small cell lung cancer cell line. PLoS ONE.

[29] Cao L, Shao M, Schilder J (2012). Tissue transglutaminase links TGF-beta, epithelial to mesenchymal transition and a stem cell phenotype in ovarian cancer. Oncogene.

[30] Kong D, Li Y, Wang Z (2011). Cancer stem cells and epithelial-to-mesenchymal transition (EMT)-phenotypic cells: are they cousins or twins?. Cancers.

[31] Shen R, Ye Y, Chen L (2008). Precancerous stem cells can serve as tumor vasculogenic progenitors. PLoS ONE.

[32] Wang R, Chadalavada K, Wilshire J (2010). Glioblastoma stem-like cells give rise to tumour endothelium. Nature.

[33] Bjerkvig R, Johansson M, Miletic H (2009). Cancer stem cells and angiogenesis. Semin Cancer Biol.

[34] Dong J, Zhao Y, Huang Q (2011). Glioma stem/progenitor cells contribute to neovascularization *via* transdifferentiation. Stem Cell Rev.

[35] Monzani E, La Porta CA (2008). Targeting cancer stem cells to modulate alternative vascularization mechanisms. Stem Cell Rev.

[36] Salmaggi A, Boiardi A, Gelati M (2006). Glioblastoma-derived tumorospheres identify a population of tumor stem-like cells with angiogenic potential and enhanced multidrug resistance phenotype. Glia.

[37] Ricci-Vitiani L, Pallini R, Biffoni M (2010). Tumour vascularization *via* endothelial differentiation of glioblastoma stem-like cells. Nature.

