# Supplementary figures and images for: Slug promoted vasculogenic mimicry in hepatocellular carcinoma
Source: J Cell Mol Med. 2013 Jul 1;17(8):1038–47. doi: 10.1111/jcmm.12087 (PMC3780534; doi:10.1111/jcmm.12087)

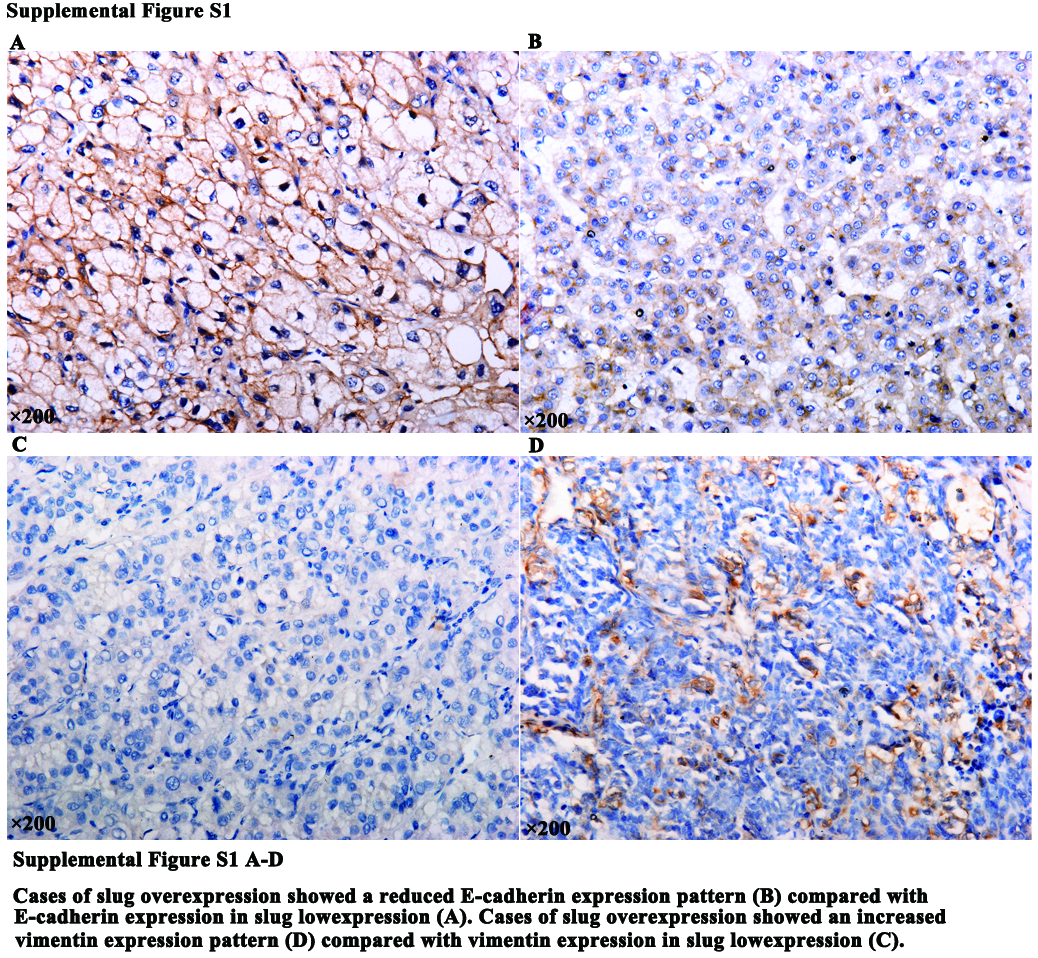

Supplement: Supplementary file 1 [file jcmm0017-1038-SD1.tif]

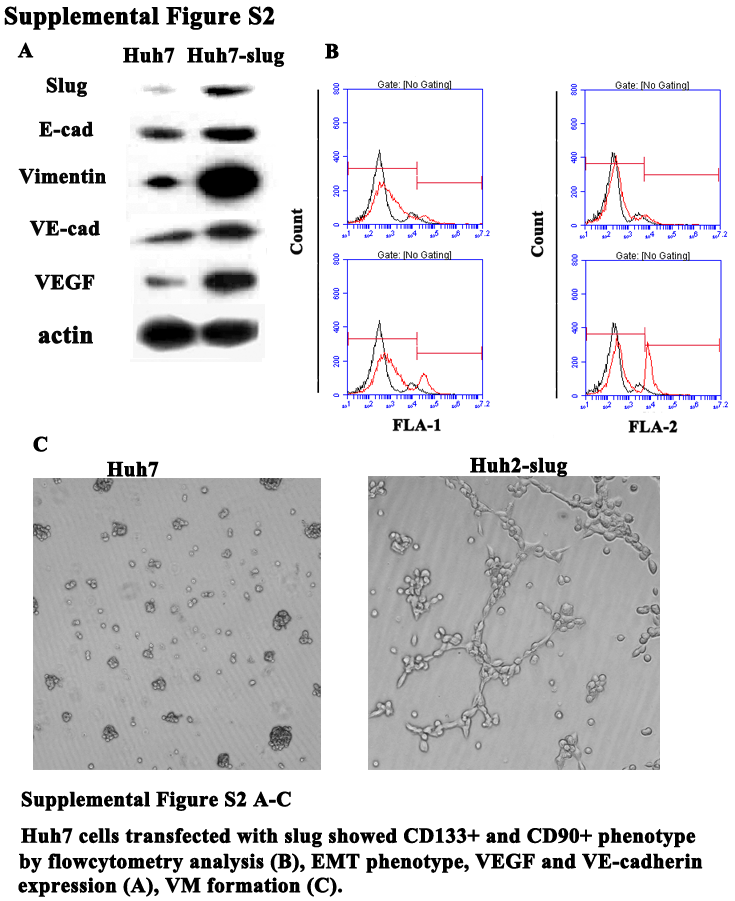

Supplement: Supplementary file 2 [file jcmm0017-1038-SD2.tif]

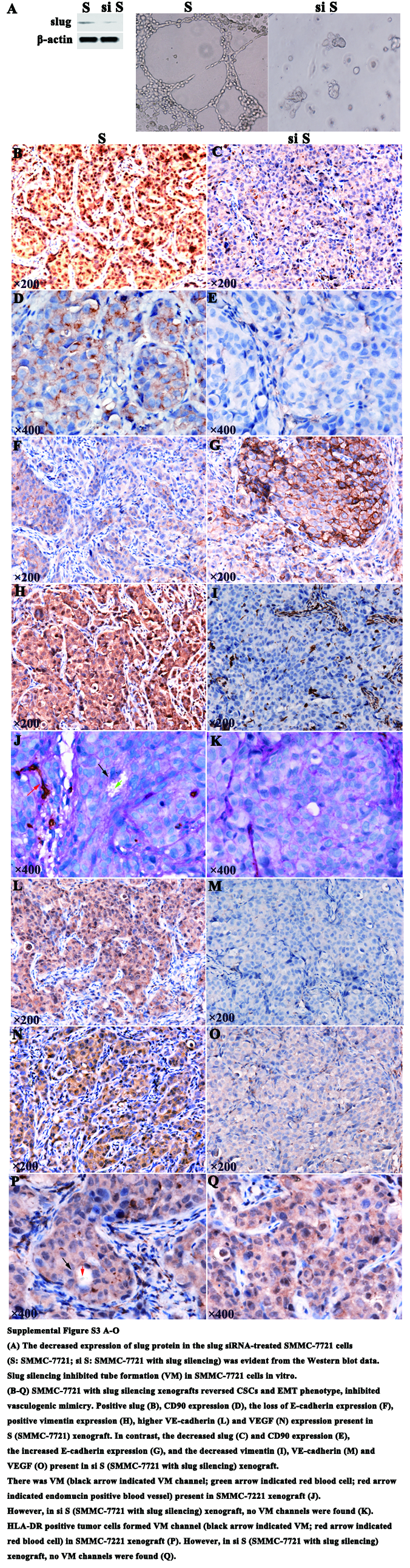

Supplement: Supplementary file 3 [file jcmm0017-1038-SD3.tif]
