# Supplementary material for: Slug promoted vasculogenic mimicry in hepatocellular carcinoma
Source: J Cell Mol Med. 2013 Jul 1;17(8):1038–47. doi: 10.1111/jcmm.12087 (PMC3780534; doi:10.1111/jcmm.12087)
Supplement: Supplementary file 4 [file jcmm0017-1038-SD4.docx]

**Supplemental data**

**Material and methods**

**Western Blot Analysis**

The whole cell lysates were resolved by way of sodium dodecyl sulfate-polyacrylamide gel electrophoresis and transferred onto polyvinylidene difluoride membranes (Millipore). Blots were blocked and incubated with the primary antibody slug (cell signaling), CD133 (Santa Cruz), VE-cadherin (Abcam), E-cadherin (Santa Cruz), vimentin (Epitimics) and VEGF (Beijing Zhongshan) followed by incubation with a secondary antibody (Santa Cruz). Blots were developed using an enhanced chemiluminescence detection kit (Amersham Pharmacia Biotech, Piscataway, NJ). For protein loading analyses, a monoclonal beta-actin antibody (Santa Cruz) was used.

**Immunofluorescence**

Cells were fixed in absolute methanol at -20°C for 20 min, blocked in 5% normal goat serum and incubated with primary antibodies. Primary antibodies against the proteins VE-cadherin (Abcam), E-cadherin (Santa Cruz), vimentin (Epitimics) and VEGF (Beijing Zhongshan) were followed by secondary antibodies. The fluorescein isothiocyanate (FITC)-conjugated mouse and rabbit immunoglobulin G antibody (Santa Cruz Biotechnology) were used as labels for immunofluorescence assay. After immunolabeling, cells were washed, stained with DAPI (Sigma), mounted, and then viewed with fluorescent microscopy (Nikon, Japan) and analyzed as maximum projections. Values reported represent the average of 100 cells in each of two independent experiments.

**Retrovirus vectors and infections**

For siRNA-mediated inhibition, the siRNA sequences against human Slug (5′-CAGACCCATTCTGATGTAAAG-3′) were cloned into the psiHIV-nH1 lentiviral vector system. Amphotropic retroviruses were created by transient cotransfection of DNA into 293T cells. 10-cm dish were cotransfected with 2.5 μg of appropriate packaging plasmid ( HPK-LvTR, GeneCopoeia) and 15μl EndoFectin Lenti. Viral supernatants were harvested at 48hrs post-transfection, passed through a 0.45μm filter, and supplemented with 8 μg/ml polybrene prior to infection.

**Supplemental Figure Legends**

**Supplemental Figure S1 A-D**

Cases of slug overexpression showed a reduced E-cadherin expression pattern (B) compared with E-cadherin expression in slug lowexpression (A). Cases of slug overexpression showed an increased vimentin expression pattern (D) compared with vimentin expression in slug lowexpression (C).

**Supplemental Figure S2 A-C**

Huh7 cells transfected with slug showed CD133+ and CD90+ phenotype by flowcytometry analysis (B), EMT phenotype, VEGF and VE-cadherin expression (A), VM formation (C).

**Supplemental Figure S3 A-O**

(A) The decreased expression of slug protein in the slug siRNA-treated SMMC-7721 cells (S: SMMC-7721; si S: SMMC-7721 with slug silencing) was evident from the Western blot data. Slug silencing inhibited tube formation (VM) in SMMC-7721 cells in vitro. (B-Q) SMMC-7721 with slug silencing xenografts reversed CSCs and EMT phenotype, inhibited vasculogenic mimicry. Positive slug (B), CD90 expression (D), the loss of E-cadherin expression (F), positive vimentin expression (H), higher VE-cadherin (L) and VEGF (N) expression present in S (SMMC-7721) xenograft. In contrast, the decreased slug (C) and CD90 expression (E), the increased E-cadherin expression (G), and the decreased vimentin (I), VE-cadherin (M) and VEGF (O) present in si S (SMMC-7721 with slug silencing) xenograft. There was VM (black arrow indicated VM channel; green arrow indicated red blood cell; red arrow indicated endomucin positive blood vessel) present in SMMC-7221 xenograft (J). However, in si S (SMMC-7721 with slug silencing) xenograft, no VM channels were found (K). HLA-DR positive tumor cells formed VM channel (black arrow indicated VM; red arrow indicated red blood cell) in SMMC-7221 xenograft (P). However, in si S (SMMC-7721 with slug silencing) xenograft, no VM channels were found (Q).
